# Laparoscopic Removal of Multiple Ingested Magnets Using the Appendix and Meckel’s Diverticulum: A Case Report

**DOI:** 10.7759/cureus.96949

**Published:** 2025-11-16

**Authors:** Félix Omoregbee, Dávid Kováts

**Affiliations:** 1 Department of Pediatric Surgery, Bethesda Gyermekkórház, Budapest, HUN

**Keywords:** appendectomy, children, laparoscopy, magnet ingestion, meckel's diverticulum

## Abstract

There has been a marked increase over the last two decades in pediatric cases involving ingestion of multiple magnets, which can cause serious, potentially life-threatening gastrointestinal complications. More than half of such incidents require surgery, and nearly three-quarters are managed via laparotomy. Magnet extraction through laparoscopic appendectomy has been described as a viable, minimally invasive method in selected cases. We report the case of a seven-year-old boy with a staggered ingestion of two magnetic spheres. The initial abdominal radiograph showed the magnets in separate locations. An urgent endoscopic retrieval attempt was unsuccessful, and subsequent radiographs demonstrated that the magnets adhered to each other in a fixed position. Diagnostic laparoscopy revealed the magnets causing an adhesion between the caecal wall and the ileum with signs of imminent perforation. The magnet located in the cecum was removed via appendectomy. Inspection of the small bowel loops revealed a Meckel’s diverticulum. The second magnet was guided into the diverticulum, secured with an endoclip, and the diverticulum containing the magnet was then exteriorized through the umbilicus and resected. This report highlights the effectiveness of appendectomy in the removal of ingested magnets. It also underscores the importance of thoroughly inspecting the small bowel during laparoscopy, as unexpected anatomical findings (such as a Meckel’s diverticulum) may influence the surgical strategy.

## Introduction

The incidence of magnetic foreign body (MFB) ingestion in children has been rising worldwide in the last two decades, largely due to the increasing availability of powerful neodymium toys [1-3]. Among these cases, special attention should be given to multiple MFB ingestion. When more than one magnet (or a magnet and another metallic object) is swallowed, their strong mutual attraction across bowel loops can cause pressure necrosis, perforation, entero-enteric fistula (EEF) formation, or obstruction. While endoscopic retrieval should be attempted when feasible, multiple MFB ingestions require operative management in the majority of cases [3]. According to recent literature, only about one-quarter of reported cases of multiple magnet ingestion were managed with a completely laparoscopic approach [4-6]. Only a few case reports have described the use of laparoscopic appendectomy for magnet retrieval [7-9]. We present a unique case in which both the appendix and a Meckel’s diverticulum were utilized laparoscopically for safe magnet retrieval.

## Case presentation

**Figure 1:**
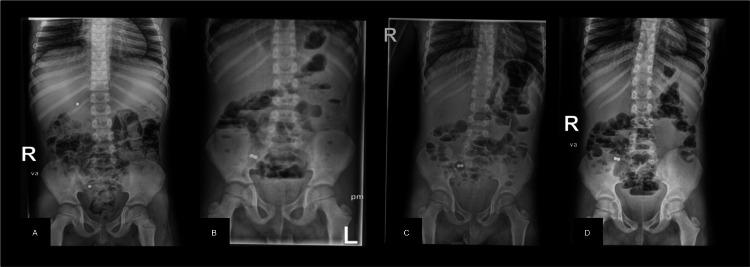
Abdominal radiographs at presentation and during observation. (A) Two magnetic foreign bodies (MFBs) seen separately on the initial radiograph. B) MFBs adhered to each other in the right lower quadrant. C–D) Serial radiographs obtained 8 and 24 hours later showed no progression of the MFBs.

A seven-year-old boy presented to the pediatric emergency department late at night after swallowing two small magnetic balls during play earlier that afternoon, approximately two hours apart. He was asymptomatic, and on examination, his abdomen was soft and non-tender, with no abnormalities detected. The initial abdominal X-ray revealed two identical, 5-mm-sized magnetic balls, one located at the level of the twelfth thoracic vertebra and the other in the right hypochondrium (Figure 1A).

The patient was admitted to the internal medicine ward, and the on-call gastroenterologist was consulted, who recommended urgent endoscopy to remove at least one magnet and prevent potential complications. At midnight, esophagogastroduodenoscopy (EGD) was performed, reaching the proximal duodenum without evidence of MFBs in the stomach or duodenum. A colonoscopy was subsequently performed without formal bowel preparation, allowing advancement up to the splenic flexure; however, fluoroscopy localized one magnet within the cecal region. Endoscopic removal was therefore unsuccessful. The following morning, abdominal radiography demonstrated that both magnets adhered to each other in the right iliac fossa (Figure 1B). Over the next 24 hours, the patient remained asymptomatic under observation and bowel prep (Picoprep, Ferring Pharmaceuticals, Germany). However, serial abdominal radiographs showed no progression of the MFBs (Figures 1C-1D). The patient was referred to pediatric surgery for evaluation, and due to the risk of perforation, diagnostic laparoscopy was indicated.

After establishing pneumoperitoneum through a 10 mm umbilical port, two additional 5 mm ports were placed, one suprapubic and one in the left mesogastrium. Initial laparoscopic exploration revealed one magnet within a loop of ileum adherent to the other magnet in the cecum. Using the magnetic attraction between the metallic tips of our instruments and the magnets, we could separate the bowels, revealing signs of imminent perforation (Video 1).

**Video 1:** Laparoscopic demonstration of magnetic attraction between the instruments and the ingested MFBs. MFB: magnetic foreign body

The magnet located in the cecum was maneuvered into the appendiceal lumen, and following placement of polymer locking clips (Hem-o-lok, Teleflex Medical, USA), a standard appendectomy was performed. The appendix containing one magnet was retrieved through the umbilical port.

At this stage, the question arose whether the removal of the second magnet was necessary. Our opinion was that it was not strictly required, as the indication for surgery (the risk of bowel wall injury from compression between two adjacent MFBs) had already been eliminated with the removal of one magnet by appendectomy. The remaining magnet was expected to pass spontaneously.

**Figure 2:**
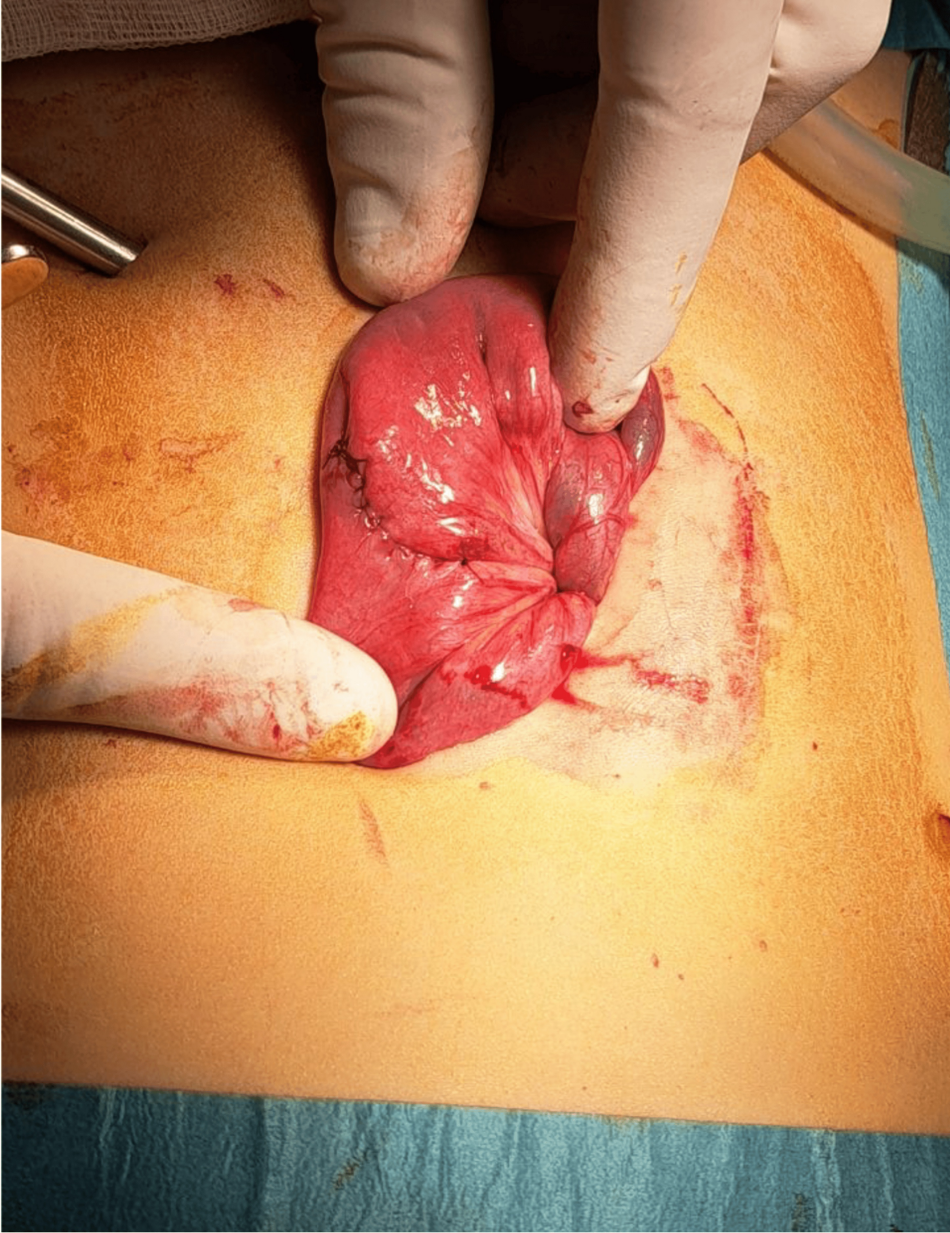
Sutured ileum after wedge resection of the Meckel’s diverticulum containing the second magnet.

Before abdominal closure, the small bowel was systematically examined retrograde from the terminal ileum to exclude any occult bowel injury related to the magnets. During inspection, a Meckel’s diverticulum was identified, providing a straightforward opportunity for the removal of the second magnet. The magnet was guided into the diverticulum, secured with a clip (Video 2), and the diverticulum was exteriorized through the umbilicus for wedge resection (Figure 2).

**Video 2:** Intraoperative video showing the magnet being guided into the Meckel’s diverticulum and secured with an endoclip before exteriorization and resection.

**Figure 3:**
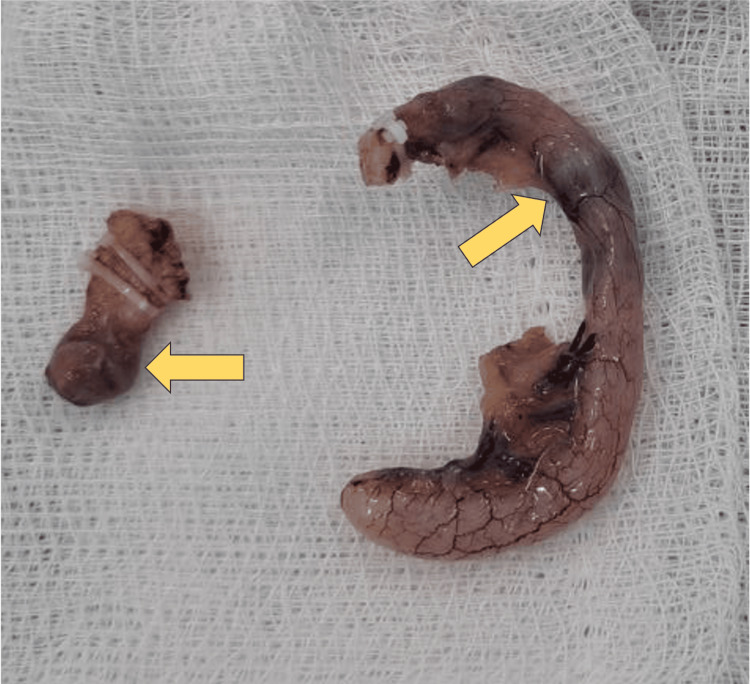
Removed Meckel’s diverticulum (left) and appendix (right) containing 1-1 magnetic foreign bodies (arrows).

Following successful extraction of the MFBs (Figure 3), the postoperative course was uneventful, and the patient was discharged on postoperative day three.

## Discussion

Multiple MFB ingestion is a well-recognized and potentially life-threatening emergency in children. The growing popularity of small, powerful neodymium magnets has led to an increasing number of emergency presentations worldwide [1, 2]. Because symptoms are often absent or nonspecific, diagnosis relies heavily on careful history and imaging. A detailed anamnesis should always consider the possibility of unwitnessed ingestion, especially in toddlers and preschool-aged children, who are often unable to provide a reliable history [3].

Chest and plain abdominal radiography is the initial imaging of choice as it reliably identifies the number, configuration, and anatomical location of MFBs [2]. Serial imaging is essential to detect progression, clustering of multiple objects, and to identify potential complications such as obstruction or free air indicating perforation [10]. Ultrasound can complement radiography to identify secondary findings such as free fluid, bowel wall thickening, or localized inflammation. Bucci et al. reported a case of multiple MFB ingestion in which ultrasound, performed after an equivocal X-ray, accurately demonstrated the MFBs’ location in different intestinal segments and was pivotal for appropriate surgical management [11].

Several management algorithms for pediatric multiple MFB ingestion have been proposed [1, 2, 10, 12-18]. All emphasize early diagnosis, risk stratification, and timely intervention to prevent complications. The current ESPGHAN position paper underscores the need to immediately distinguish between single and multiple magnet ingestions, with particular attention to staggered ingestion. It recommends early multidisciplinary management involving pediatric surgery and gastroenterology, and prioritizes urgent endoscopic or surgical removal when multiple magnets are beyond the stomach or when the number ingested is uncertain. Conservative management is reserved for only selected, closely monitored cases [2].

Recent retrospective multicenter and institutional studies confirm that in children, the ingestion of MFBs is associated with a high rate of surgical intervention and intestinal perforation [3, 15-17, 19]. The number of magnets ingested has been shown to correlate directly with surgical morbidity [2, 15]. In a systematic review, Hayward and Saxena analyzed operative approaches and surgical outcomes for multiple MFB ingestions. Among 136 pediatric cases, laparoscopic management resulted in shorter hospital stays and fewer postoperative complications than open surgery, supporting minimally invasive methods as the preferred option when feasible [4]. Kelaidi et al.’s recent systematic review focused on EEF following ingestions of MFBs, compiling 130 pediatric cases. They found that nearly 96% of EEF cases required operative intervention (most commonly for ileal or jejunal fistulas) and were associated with a 19% postoperative complication rate [20].

Few case reports have reported the deliberate use of the appendix for ingested magnet extraction. The Magnet Extraction Through Appendectomy Laparoscopically (METAL) technique, described by Sun et al., used laparoscopic instruments to guide magnets into the appendiceal lumen, followed by standard appendectomy [8]. Dotlacil et al. reported two cases in which MFBs were successfully removed by laparoscopic appendectomy, and Gizewska-Kacprzak et al. reported similar surgical management in two teenagers, highlighting the use of polymer clips and avoiding coagulation [7, 9]. In all cases, patients recovered uneventfully. Schaffer et al. proposed the Early Colonic Preparation and Salvage Laparoscopic Appendectomy (ECSLA) protocol, combining early colonic preparation with either colonoscopic retrieval or “salvage” laparoscopic appendectomy, achieving complete recovery in all reported cases without laparotomy [18].

Our case adds to the growing evidence that the appendix provides a simple, low-risk route for the extraction of MFBs from the small bowel. Laparoscopic appendectomy avoids enterotomy, which would otherwise require a larger midline incision and also carry a higher risk of contamination, leakage, and postoperative complications [8]. Systematic inspection of the entire small bowel is essential, as mucosal injuries or even EEFs may be overlooked. In our patient, this exploration revealed a Meckel’s diverticulum, which offered a unique and practical solution for removing the remaining magnet. To the best of our knowledge, this is the first reported case in which a Meckel’s diverticulum was deliberately utilized to retrieve a foreign body.

## Conclusions

The incidence of multiple magnet ingestions is rising globally. Adherence to established guidelines and early multidisciplinary management involving gastroenterology and pediatric surgery are essential. When endoscopic removal fails, laparoscopy provides a reliable method for both evaluation and treatment. In selected cases, laparoscopic magnet extraction through appendectomy is a safe and effective method. Thorough inspection of the bowel to identify occult injuries or retained magnets is mandatory. The optimal surgical approach for magnet removal should be determined on a case-by-case basis after careful laparoscopic assessment of the extent of injury and any anatomical findings that may influence surgical planning.
